# Sarcopenia as a predictor of cage subsidence following stand-alone oblique lumbar interbody fusion in non-osteoporotic patients

**DOI:** 10.3389/fsurg.2026.1763893

**Published:** 2026-02-17

**Authors:** Dazhuang Miao, Xianda Gao, Weiqi Zhang, Xiaowei Ma, Di Zhang

**Affiliations:** The Spine Surgery Department of the Third Hospital of Hebei Medical University, Shijiazhuang, China

**Keywords:** bone mineral density, cage subsidence, predictors, sarcopenia, stand-alone oblique interbody fusion

## Abstract

**Objective:**

This study aimed to identify predictors of cage subsidence following stand-alone oblique interbody fusion (SA-OLIF) in non-osteoporotic patients.

**Methods:**

A retrospective analysis was performed on 98 patients who underwent SA-OLIF. Cage subsidence was defined to have occured when a cage was subsided into the adjacent endplate by more than 2 mm on the last follow up radiographs. Patients were categorized into subsidence and non-subsidence groups accordingly. Patient characteristics, radiographic parameters, and clinical outcomes were recorded. Sarcopenia was assessed using the L3 skeletal muscle index on axial computed tomography images. Multivariate logistic regression analysis was conducted to identify the predictors of cage subsidence following SA-OLIF.

**Results:**

Of the 98 patients who underwent SA-OLIF, subsidence occurred in 32 (32.7%). The subsidence group had a higher mean age (*P* = 0.005) and lower bone mineral density (BMD) (*P* < 0.001). The prevalence of sarcopenia was significantly greater in the subsidence group compared with the non-subsidence group (*P* = 0.003). Multivariate logistic regression identified sarcopenia (*P* = 0.021), age (*P* = 0.011), and BMD (*P* < 0.001) as predictors of cage subsidence. The areas under the curve for age and BMD in predicting cage subsidence were 0.676 and 0.783, respectively.

**Conclusion:**

Cage subsidence following SA-OLIF was a common complication in non-osteoporotic patients, with an incidence rate of 32.7%. Preoperative sarcopenia, age of >59.5 years, and T-score < −1.9 were predictors of cage subsidence following SA-OLIF in non-osteoporotic patients. Patients with sarcopenia had nearly 4-fold increased odds of subsidence. OLIF with instruments might be considered an alternative surgical method for patients with these predictor factors to decrease the incidence of cage subsidence.

## Introduction

The term “sarcopenia” was first proposed by Irwin Rosenberg in 1989 and refers to the progressive loss of muscle mass, strength, and function in the elderly (1, 2). The etiology of sarcopenia is multifactorial, with reduced physical activity, inadequate nutrition, disease triggers, activation of inflammatory pathways, loss of neuromuscular junction integrity, and hormonal changes all contributing (1). Sarcopenia has been associated with adverse outcomes in several conditions, including chronic obstructive pulmonary disease (3), coronary heart disease (4), and cancer (5). Recent studies have also indicated that patients with sarcopenia are more likely to experience poor outcomes following spinal surgery (6). However, the relationship between sarcopenia and cage subsidence following stand-alone oblique lumbar interbody fusion (SA-OLIF) has not yet been investigated.

Oblique lumbar interbody fusion (OLIF), a minimally invasive surgical approach, was first proposed by Mayer in 1997 (7). SA-OLIF and OLIF with instrumentation are two surgical strategies for treating lumbar spine disorders (8–12). SA-OLIF offers several advantages, including shorter operative time, reduced intraoperative bleeding, fewer implant-related complications, lower treatment costs, and shorter hospital stays (10). Nevertheless, cage subsidence remains a common complication following SA-OLIF, making the identification of appropriate surgical indications a critical issue.

Our previous study demonstrated that the most common clinical symptom of cage subsidence was low back pain (13), which was relieved by conservative treatment in most cases. However, severe cage subsidence can induce spinal stenosis and nerve compression, necessitating revision surgery. Osteoporosis [bone mineral density (BMD) T-score < −2.5] has been established as a significant risk factor for cage subsidence, for which instrumentation is recommended (14–16). However, cage subsidence is also frequently observed in for patients with *T*-score higher than −2.5 following SA-OLIF. Therefore, the present study aimed to identify predictors of cage subsidence following SA-OLIF in non-osteoporotic patients, providing evidence to guide surgical planning in clinical practice.

## Materials and methods

### Patients

This study was approved by the Clinical Ethics Committee of the Third Affiliated Hospital of Hebei Medical University. In accordance with national legislation and institutional requirements, written informed consent was not required for participation.

A retrospective review was conducted on 98 patients who underwent SA-OLIF between January 2018 and January 2023 at the Third Hospital of Hebei Medical University, China. Inclusion criteria were as follows: (1) patients with symptomatic lumbar degenerative diseases (Grade I lumbar spondylolisthesis, lumbar instability, mild to moderate spinal stenosis, or degenerative lumbar disc disease) who had not improved after at least 3 months of conservative treatment; (2) clinical symptoms consistent with magnetic resonance imaging (MRI) and computed tomography (CT) findings; (3) single-level SA-OLIF; and (4) minimum follow-up of 2 years. Exclusion criteria were as follows: (1) previous spinal fusion surgery; (2) tumors, inflammation, trauma, or other spinal diseases; (3) osteoporosis (*T*-score < −2.5); (4) incomplete medical records; and (5) extremely high body mass index (BMI) or intraoperative endplate injury.

### Surgical procedure

All procedures were performed by the same experienced surgical team. Under general anesthesia, patients were placed in a standard right lateral position and secured with tape. The C-arm X-ray machine was used to mark the center of the target intervertebral disc space, after which a 4-cm incision was made medial to the marked point. The skin, subcutaneous tissue, and external oblique aponeurosis were incised, and blunt dissection was performed to separate the fibers of the external oblique, internal oblique, and transversus abdominis muscles. Access to the disc space was achieved through the retroperitoneal corridor between the psoas major and the great vessel sheath, with careful identification and protection of the genitofemoral nerve, peritoneum, great vessel sheath, ureters, and any segmental vessels. Once the surgical intervertebral space was confirmed, a fixed working channel was installed. The annulus fibrosus was incised, and the nucleus pulposus and cartilaginous endplate tissues above and below the disc were carefully removed, avoiding injury to the bony endplate. A suitable polyetheretherketone cage (6 °Clydesdale; Medtronic Sofamor Danek, Memphis, Tennessee, USA) filled with allogeneic trabecular bone and demineralized bone matrix was implanted under intraoperative C-arm fluoroscopic guidance. On the third postoperative day, patients were encouraged to ambulate with waist support and to perform low back muscle exercises under professional guidance.

### Sarcopenia assessment

Sarcopenia was assessed in accordance with the diagnostic criteria recommended by the European Working Group on Sarcopenia in Older People (EWGSOP) (17, 18). The L3 skeletal muscle index (L3-SMI) was measured on axial computed tomography images, although this cannot fully reflect physical performance. The L3-SMI was calculated as the skeletal muscle area at the level of the third lumbar vertebra divided by the square of the patient's height (cm^2^/m^2^). Sarcopenia was defined using an L3-SMI cutoff of 45.4 cm^2^/m^2^ for male patients and 34.4 cm^2^/m^2^ for female patients (18).

### Radiological and clinical evaluation

Patients were categorized into subsidence and non-subsidence groups. Preoperative variables including age, sex, BMI, BMD, smoking status, symptom duration, operative level, and fusion rate were recorded. Dual-energy X-ray absorptiometry was used to assess BMD, with values below −2.5 defined as osteoporosis.

**Figure 1:**
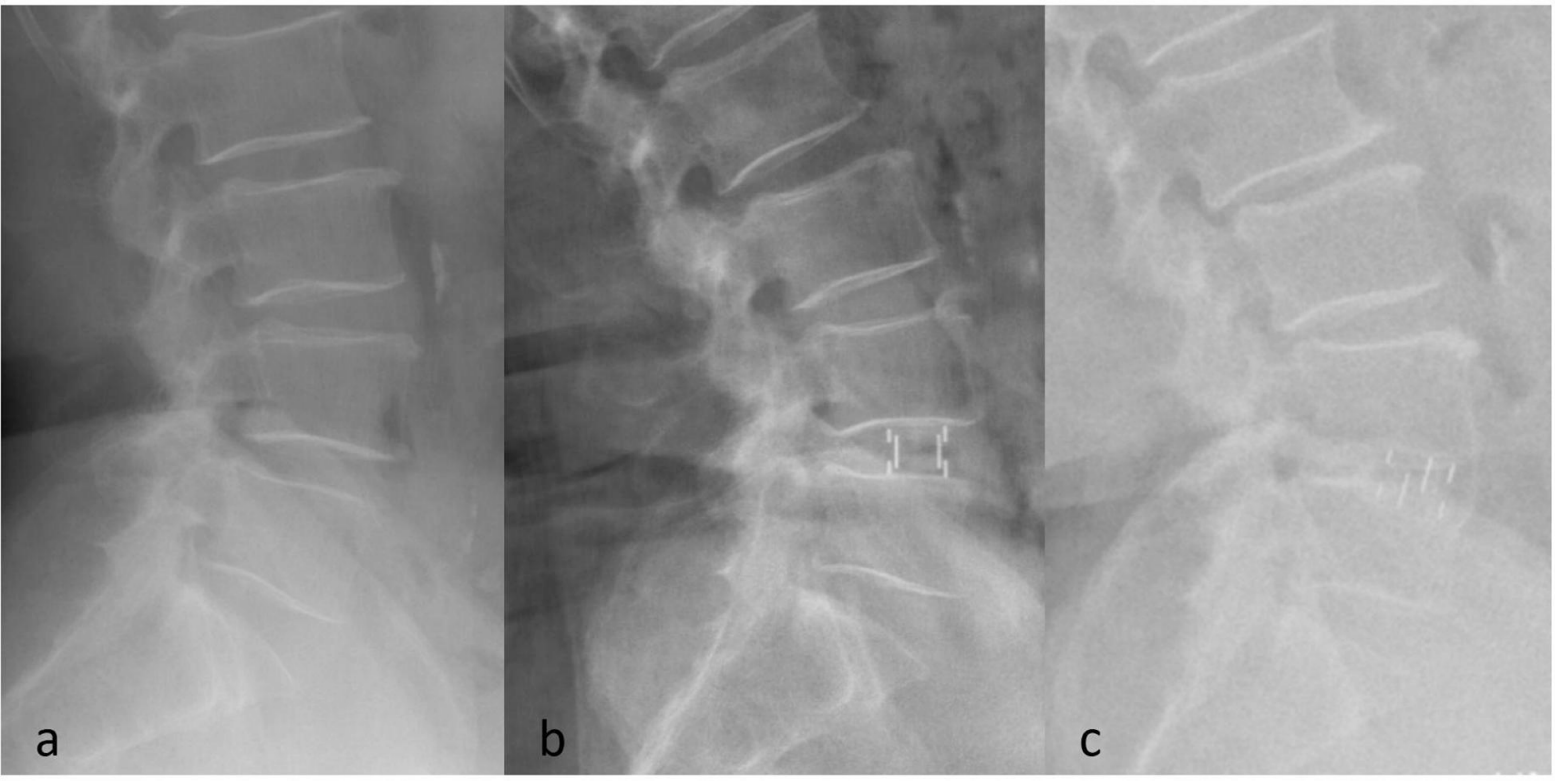
Preoperative **(a)**, postoperative **(b)**, and last follow-up **(c)** radiographs of SA-OLIF.

Radiological outcomes were evaluated using radiographs, CT, and MRI preoperatively, postoperatively, and at the last follow-up. Lumbar lordosis (LL) was measured as the Cobb angle between the superior endplate of L1 and the inferior endplate of L5. Local lordosis was defined as the Cobb angle between the superior endplate of the upper vertebra and the inferior endplate of the lower vertebra at the surgical segment. Intervertebral height (IH) was calculated as the average of the anterior, middle, and posterior heights between adjacent endplates. ΔIH was defined as the difference between postoperative and last follow-up IH; a ΔIH greater than 2 mm was diagnosed as cage subsidence (Figure 1) (19). All imaging measurements were independently performed by two spinal surgeons, with disagreements resolved by a third senior surgeon. Measurements were obtained using the DICOM-PACS program.

Clinical outcomes were assessed using questionnaires administered preoperatively and at the final follow-up visit. Lumbar function and pain intensity were evaluated using the Oswestry Disability Index (ODI; 0–50), with postoperative low back pain defined as a final ODI score exceeding the preoperative score. Neurological function was assessed using the Japanese Orthopaedic Association (JOA) score (0–29), and the recovery rate was calculated as follows: recovery rate (%) = (final JOA score−preoperative JOA score)/(29−preoperative JOA score) × 100%.

### Statistical analysis

Data were analyzed using SPSS software (version 27.0; IBM, Armonk, NY, USA), with *p*-values <0.05 considered statistically significant. The Shapiro–Wilk test was used to assess normality. Quantitative data between groups were compared using the independent-samples *t*-test or Mann–Whitney *U*-test, as appropriate. Paired-sample *t*-tests or Wilcoxon tests were used to compare preoperative and final follow-up values within groups, including LL and local lordosis. Friedman analysis was applied for repeated measures of IH across preoperative, postoperative, and final follow-up assessments; *p*-values were adjusted to 0.0167 using Bonferroni correction for multiple comparisons. Categorical data were compared using the Chi-square or Fisher's exact test, as appropriate. Univariate logistic regression was used to identify potential predictors (*P* < 0.100) for inclusion in the multivariate logistic model. Multivariate logistic regression was performed, with results expressed as adjusted odds ratios (ORs) and 95% confidence intervals (CIs). Variables with *p* < 0.05 were considered significant predictors of cage subsidence following SA-OLIF. The sensitivity, specificity, and optimal cutoff values for each predictor were determined by receiver operating characteristic (ROC) curve analysis, selecting the value corresponding to the maximum Youden index.

Sample size was calculated using PASS 15 (version 15; NCSS LLC, Kaysville, UT, USA). A pilot study including 55 patients meeting the inclusion and exclusion criteria was conducted. Sarcopenia, age, and sex were included in the multivariate logistic regression, yielding an OR of 4.141 for sarcopenia. With an expected effect size of 4.141, a desired statistical power of 0.8, and an alpha of 0.05, the minimum sample size was calculated as 96. Therefore, 98 subjects were included, meeting this threshold.

## Results

Among the 98 patients included, 32 (32.7%) met the subsidence criteria and were classified as the subsidence group, while the remaining 66 (67.3%) constituted the non-subsidence group. Patient characteristics are presented in Table 1. In the subsidence group, 15 patients (46.9%) were diagnosed with sarcopenia, with a mean L3-SMI of 38.64 ± 6.83 cm^2^/m^2^. In the non-subsidence group, 12 patients (18.2%) were diagnosed with sarcopenia, with a mean L3-SMI of 43.71 ± 8.35 cm^2^/m^2^, representing a significant difference between groups (*P* = 0.003 and *P* = 0.001, respectively). Patients in the subsidence group were significantly older than those in the non-subsidence group (*P* = 0.005). The subsidence group also had lower BMD compared with the non-subsidence group (−2.05 ± 0.31 vs. −1.61 ± 0.43, *P* < 0.001). There were no significant differences between groups regarding BMI (23.93 ± 2.54 vs. 24.72 ± 3.06, *P* = 0.102), smoking status (15.6% vs. 10.6%, *P* = 0.702), surgical segment (*P* = 0.187), or fusion rate (90.6% vs. 93.9%, *P* = 0.858).

**Table 1:** Comparison of patient characteristics between subsidence group and non-subsidence group.

Variable	Subsidence group (*n* = 32)	Non-subsidence group (*n* = 66)	*t/z/χ* ^2^	*P*-value
Age (years)	63.28 ± 5.87	58.52 ± 7.50	2.822	0.005
Gender				
Male	7	10	0.679	0.410
Female	25	56		
Body mass index	23.93 ± 2.54	24.72 ± 3.06	1.649	0.102
BMD (*T*-score)	−2.05 ± 0.31	−1.61 ± 0.43	4.548	<0.001
Smoking	5	7	0.146	0.702
History of symptoms (months)	11.81 ± 6.95	12.62 ± 9.57	0.186	0.853
Followed up period (months)	26.69 ± 4.75	26.24 ± 4.65	0.656	0.512
L3-SMI (cm^2^/m^2^)	38.64 ± 6.83	43.71 ± 8.35	3.189	0.001
Sarcopenia	15	12	8.889	0.003
Surgical segment				
L2/3	3	2	3.120	0.187*
L3/4	9	13		
L4/5	20	51		
Fusion rate%	90.6% (29/32)	93.9% (62/66)	0.032	0.858
Revision rate%	0	0		

BMD, bone mineral density; SMI, skeletal muscle mass index.

* Fisher's exact test.

The radiological and clinical outcomes are presented in Table 2. In both groups, last follow-up LL and local lordosis Cobb angle were significantly increased compared with preoperative values (*P* < 0.001). In the non-subsidence group, the last follow-up IH was statistically greater than preoperative IH (10.80 ± 1.63 vs. 9.01 ± 1.73 mm, *P* < 0.001). In contrast, in the subsidence group, there was no significant difference between last follow-up IH and preoperative IH (9.44 ± 1.53 vs. 8.84 ± 1.66 mm, *P* = 0.401). There were no significant differences in IH between groups preoperatively or postoperatively. Last follow-up IH in the subsidence group was lower than in the non-subsidence group (10.80 ± 1.63 vs. 9.44 ± 1.53 mm, *P* < 0.001). ΔIH was 0.91 ± 0.60 mm in the non-subsidence group and 2.82 ± 0.71 mm in the subsidence group, showing a significant difference (*P* < 0.001). The last follow-up local lordosis Cobb angle in the non-subsidence group was significantly higher than that in the subsidence group, and the difference was statistically significant (*P* = 0.008). No significant differences were observed in cage height or length between the two groups. Clinical outcomes, assessed using ODI and JOA scores, improved significantly in both groups at the last follow-up. There were no significant differences in JOA or ODI scores between groups preoperatively or postoperatively. Postoperative low back pain was reported in seven patients (21.9%) in the subsidence group and six patients (9.1%) in the non-subsidence group, although this difference was not statistically significant.

**Table 2:** Comparison of radiological and clinical outcomes between subsidence group and non-subsidence group.

Variable	Subsidence group (*n* = 32)	Non-subsidence group (*n* = 66)	*t/z/χ* ^2^	*P*-value
Intervertebral height (mm)				
Preoperative	8.84 ± 1.66	9.01 ± 1.73	0.468	0.641
Postoperative	12.26 ± 1.25**	11.71 ± 1.65**	1.568	0.117
Last follow-up	9.44 ± 1.53	10.80 ± 1.63**	3.913	<0.001
ΔIH	2.82 ± 0.71	0.91 ± 0.60	8.000	<0.001
Lumbar lordosis				
Preoperative	29.66 ± 9.01	32.02 ± 11.17	1.041	0.300
Last follow-up	35.56 ± 10.24*	39.20 ± 10.52*	1.645	0.100
Local lordosis Cobb angle				
Preoperative	15.13 ± 3.11	15.86 ± 4.27	1.512	0.130
Last follow-up	16.31 ± 4.21*	18.71 ± 4.67*	2.647	0.008
Cage height				
8 mm	2	3	4.284	0.115^a^
10 mm	11	38		
12 mm	18	25		
Cage length				
40 mm	2	6	3.747	0.157^a^
45 mm	19	49		
50 mm	11	11		
ODI scores				
Preoperative	26.38 ± 1.15	24.92 ± 0.95	0.853	0.393
Last follow-up	15.28 ± 1.51*	12.35 ± 0.64*	1.151	0.250
Low back pain	7	6	2.882	0.112
JOA scores				
Preoperative	17.16 ± 0.49	17.55 ± 0.29	0.735	0.463
Last follow-up	21.81 ± 0.62*	22.74 ± 0.39*	1.18	0.238
Recovery rate%	38.68 ± 4.65%	45.74 ± 2.97%	1.356	0.175

* Statistically significant difference from preoperative (*P* < 0.05).

** Statistically significant difference from preoperative (*p* < 0.0167).

^a^ Fisher's exact test.

**Figure 2:**
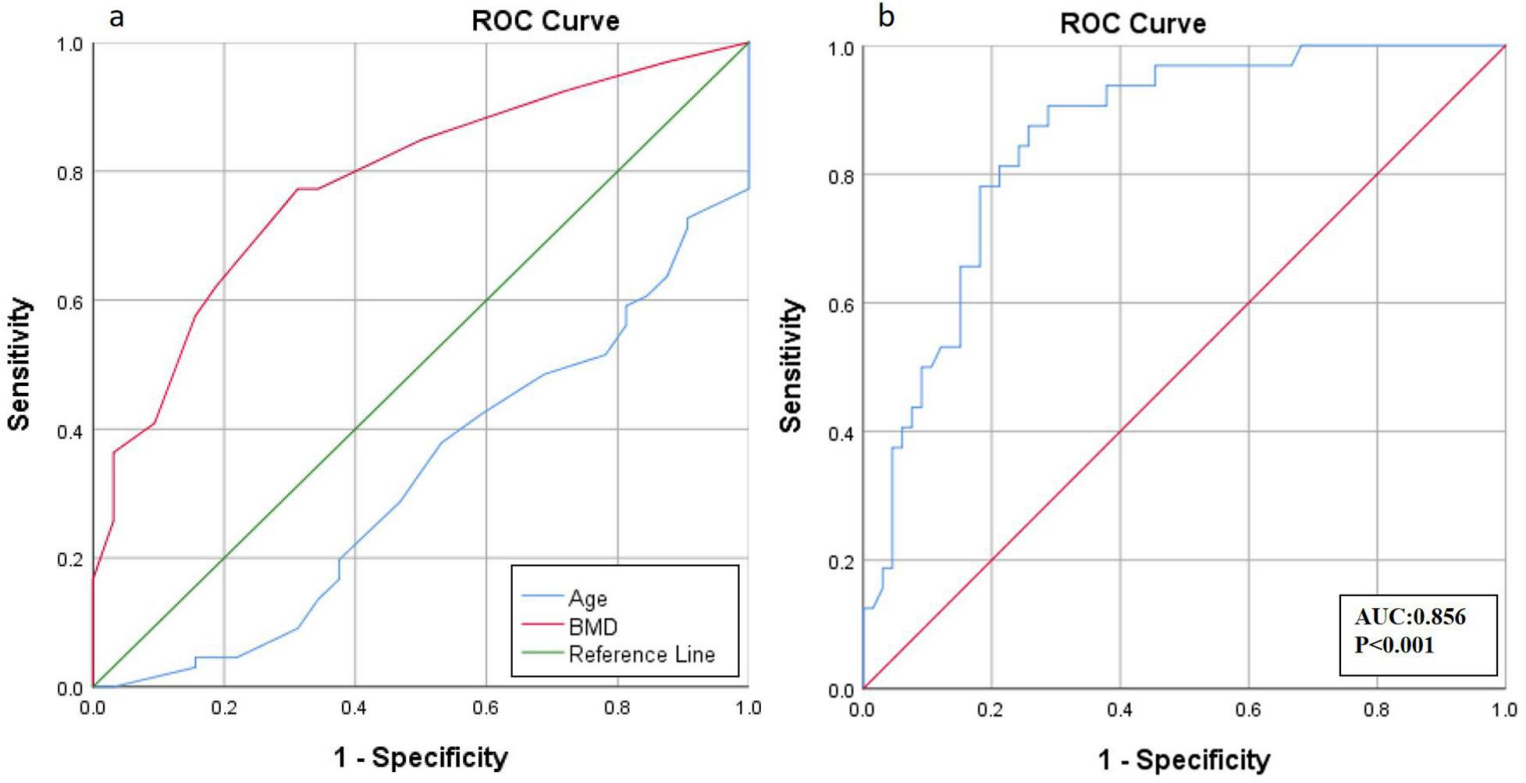
Receiver operating characteristic (ROC) curve analysis. **(a)** The cutoff values of age and BMD were 59.5 and −1.9 respectively. **(b)** The area under the curve (AUC) in regard to logistic regression model was 0.856.

Univariate analysis identified age (*P* = 0.004), BMD (*P* < 0.001), and sarcopenia (*P* = 0.004) as potential predictors for inclusion in the multivariate logistic model. Multivariate logistic regression (Table 3) revealed that age (OR = 1.113, 95% CI = 1.025–1.208, *P* = 0.011), BMD (OR = 0.059, 95% CI = 0.014–0.256, *P* < 0.001), and sarcopenia (OR = 3.881, 95% CI = 1.231–12.241, *P* = 0.021) were independent predictors of cage subsidence following SA-OLIF. ROC analysis of the multivariate model yielded an area under the curve (AUC) of 0.856 (*P* < 0.001) (Figure 2b).

**Table 3:** Multivariate logistic regression analysis of predictors for cage subsidence following SA-OLIF.

Variable	Adjusted odds radio	95% confidence interval	*P*-value
Age (years)	1.113	1.025–1.208	0.011
BMD	0.059	0.014–0.256	<0.001
Sarcopenia	3.881	1.231–12.241	0.021

BMD, bone mineral density.

ROC analysis was also performed to evaluate the predictive value of age and BMD for cage subsidence in non-osteoporotic patients, with cutoff values determined using the Youden index (Figure 2a, Table 4). The AUC for age was 0.676, with a cutoff of 59.5 years (sensitivity 78.1%, specificity 48.5%). The AUC for BMD was 0.783, with a cutoff of −1.95 (sensitivity 77.3%, specificity 68.7%).

**Table 4:** Sensitivity, specificity, AUC, and cutoff of predictors.

Variable	Sensitivity (%)	Specificity (%)	AUC^a^	Cutoff	*P*-value
Age (years)	78.1	48.5	0.676	59.5	0.005
BMD	77.3	68.7	0.783	−1.95	<0.001

BMD, bone mineral density.

^a^ Area under the curve.

## Discussion

Multiple diagnostic approaches for sarcopenia are currently available, including the measurement of muscle mass, assessment of muscle strength, evaluation of physical activity, and analysis of biomarkers (20). Among these, indicators of muscle mass and quality derived from CT are highly effective in assessing sarcopenia (21). The patients in this study were elderly with lumbar degenerative diseases and had limitations in performing functional tests, such as walking assessments, for sarcopenia evaluation. Therefore, although L3-SMI does not fully reflect physical function, it was employed as a simple and objective method to diagnose sarcopenia.

Cage subsidence is one of the most common complications following OLIF (22) and can lead to progressive spinal deformity and nerve compression (23). Currently, no standardized definition of cage subsidence exists, contributing to variability in reported subsidence rates (24). Kim et al. proposed a classification standard, defining cage subsidence as a disc height decrease of more than 2 mm, a criterion widely used in clinical practice (25). In the present study, the incidence of cage subsidence was 32.7%, consistent with previous reports by Hu et al. (26). A fundamental determinant in the occurrence of cage subsidence is the balance between the stress at the cage–vertebral endplate interface and the strength of the support at this interface. This balance is susceptible to various factors (27).

Multivariate logistic analysis in this study identified sarcopenia as an independent predictor of cage subsidence following SA-OLIF. Sarcopenia reflects malnutrition, physical disability, inactivity, increased frailty, and reduced quality of life, and has been associated with poorer surgical outcomes (28). Sun et al. retrospectively reviewed 314 patients aged 60–80 years who underwent single-segment posterior lumbar fusion and found that MRI-based central sarcopenia negatively affected visual analogue scale (VAS) scores for low back pain and ODI scores (29). Similarly, Matsuo et al. analyzed 178 patients with lumbar spinal stenosis (LSS) and reported that patients with LSS and sarcopenia exhibited greater vertebral slippage, lower BMI and BMD, reduced physical function, and more severe low back pain compared with those without sarcopenia (28). Our previous study of 116 patients also demonstrated that sarcopenic patients experienced a greater degree and higher incidence of postoperative low back pain, and the rate of cage subsidence was significantly higher in the sarcopenia group than in the non-sarcopenia group (15).

The findings of the present study support that sarcopenia is a predictor of cage subsidence. Patients with sarcopenia have weakened paraspinal musculature, and muscle dysfunction reduces the protective support of adjacent bone structures, increasing the risk of postoperative cage subsidence. With regard to the relationship between paraspinal muscles and cage subsidence, Kotheeranurak et al. identified multifidus muscle degeneration as a risk factor for cage subsidence following OLIF (22). Singhatanadgige et al. reported that when muscle degeneration occurs, the anterior spinal column, including the OLIF cage, absorbs higher compressive forces and is more prone to endplate violation and subsequent cage subsidence (30). Therefore, patients with preoperative sarcopenia are more prone to experiencing cage subsidence following SA-OLIF.

In the present study, BMD and age were also identified as independent predictors of cage subsidence following SA-OLIF. Our results showed that patients aged over 59.5 years were at higher risk of subsidence, consistent with the findings of Shen et al. (38). However, age showed only a moderate correlation with the extent of cage subsidence (AUC = 0.676). As an independent predictor, the clinical utility of age is limited due to its non-specific nature. Advanced age is often accompanied by declines in musculoskeletal quality and osteoporosis, which may be more directly captured by L3-SMI and BMD (31). Therefore, although age is a predictive factor, its modest predictive value suggests it should not serve as the sole criterion for surgical decision-making.

Previous studies (31, 32) have demonstrated that BMD decreases with age, reducing the load threshold for endplate failure and making it more susceptible to damage. A biomechanical analysis by Palepu et al. (33) found that, regardless of cage type, bone quality was strongly associated with the degree of cage subsidence. Similarly, Jones et al. reported that decreased endplate volumetric BMD was significantly correlated with an increased rate of subsidence (34). However, some reports have suggested that while BMD is the gold standard for assessing bone quality, it may be influenced by confounding factors such as spinal deformities, bowel contents, or aortic atherosclerosis, which can reduce measurement accuracy (35, 36).

In the present study, even in non-osteoporotic patients, BMD remained a significant predictor of cage subsidence following SA-OLIF. Patients with BMD between −1.9 and −2.5 exhibited insufficient vertebral endplate strength, increasing the risk of subsidence. Nonetheless, the predictive value of BMD was moderate (AUC = 0.783), consistent with previous studies. Zou et al. reported that Hounsfield units measured by quantitative computed tomography (qCT) more accurately assess bone quality than BMD (37). However, qCT is costly and exposes patients to higher radiological risks, limiting its use for routine examinations.

Given that cage subsidence is multifactorial, surgeons should consider all relevant factors to optimize outcomes. In this model, patients aged >59.5 years with low BMD (*T*-score <−1.9) and concomitant sarcopenia represent a high-risk cohort, with a strong correlation to cage subsidence (AUC = 0.856). Therefore, for those patients, instruments should be considered in the surgical plan to decrease the incidence rate of cage subsidence.

This study had several limitations. First, this was a single-center retrospective study with a relatively modest sample size, which may limit statistical power and introduce potential biases in patient selection and data collection. In future studies, a larger sample size might provide more generalizable and robust findings. Second, we only analyzed some common preoperative factors; the factors that may affect the cage subsidence after surgery need to be explored in future research. Third, although multiple methods exist for diagnosing sarcopenia according to EWGSOP guidelines, this study relied solely on L3-SMI and did not incorporate functional muscle assessments.

## Conclusion

Cage subsidence following SA-OLIF was a common complication in non-osteoporotic patients, with an incidence rate of 32.7%. Preoperative sarcopenia, age >59.5 years, and a *T*-score <−1.9 were predictors of cage subsidence following SA-OLIF in non-osteoporotic patients. Patients with sarcopenia had nearly 4-fold increased odds of subsidence. OLIF with instruments may represent an alternative surgical method for patients with predictor factors to decrease the incidence of cage subsidence.

## Data Availability

The raw data supporting the conclusions of this article will be made available by the authors without undue reservation.

## References

[1] Walston JD. Sarcopenia in older adults. Curr Opin Rheumatol. (2012) 24(6):623–7. 10.1097/BOR.0b013e328358d59b 22955023 PMC4066461

[2] Derstine BA Holcombe SA Goulson RL Ross BE Wang NC Sullivan JA Quantifying sarcopenia reference values using lumbar and thoracic muscle areas in a healthy population. J Nutr Health Aging. (2017) 21(10):180–5. 10.1007/s12603-017-0983-3 29300439 PMC12880485

[3] Nan Y Zhou Y Dai Z Yan T Zhong P Zhang F Role of nutrition in patients with coexisting chronic obstructive pulmonary disease and sarcopenia. Front Nutr. (2023) 10:1214684. 10.3389/fnut.2023.1214684 37614743 PMC10442553

[4] Zhang N Zhu WL Liu XH Chen W Zhu ML Kang L Prevalence and prognostic implications of sarcopenia in older patients with coronary heart disease. J Geriatr Cardiol. (2019) 16(10):756–63. 10.11909/j.issn.1671-5411.2019.10.002 31700515 PMC6828602

[5] Jogiat U Jimoh Z Turner SR Baracos V Eurich D Bédard ELR. Sarcopenia in lung cancer: a narrative review. Nutr Cancer. (2023) 75(7):1485–98. 10.1080/01635581.2023.2212425 37177914

[6] Gibbons D Ahern DP Curley AE Kepler CK Butler JS. Impact of sarcopenia on degenerative lumbar spondylosis. Clin Spine Surg. (2021) 34(2):43–50. 10.1097/BSD.0000000000001047 33633055

[7] Mayer HM. A new microsurgical technique for minimally invasive anterior lumbar interbody fusion. Spine (Phila Pa 1976). (1997) 22(6):691–9. 10.1097/00007632-199703150-00023 9089943

[8] Cai K Luo K Zhu J Zhang K Yu S Ye Y Effect of pedicle-screw rod fixation on oblique lumbar interbody fusion in patients with osteoporosis: a retrospective cohort study. J Orthop Surg Res. (2021) 16(1):429. 10.1186/s13018-021-02570-8 34217340 PMC8254285

[9] Fang G Lin Y Wu J Cui W Zhang S Guo L Biomechanical comparison of stand-alone and bilateral pedicle screw fixation for oblique lumbar interbody fusion surgery—a finite element analysis. World Neurosurg. (2020) 141:e204–12. 10.1016/j.wneu.2020.05.245 32502627

[10] Zhu G Hao Y Yu L Cai Y Yang X. Comparing stand-alone oblique lumbar interbody fusion with posterior lumbar interbody fusion for revision of rostral adjacent segment disease: a STROBE-compliant study. Medicine (Baltimore). (2018) 97(40):e12680. 10.1097/MD.0000000000012680 30290656 PMC6200540

[11] Huo Y Yang D Ma L Wang H Ding W Yang S. Oblique lumbar interbody fusion with stand-alone cages for the treatment of degenerative lumbar spondylolisthesis: a retrospective study with 1-year follow-up. Pain Res Manag. (2020) 2020:9016219. 10.1155/2020/9016219 32399131 PMC7201502

[12] Jin C Xie M He L Xu W Han W Liang W Oblique lumbar interbody fusion for adjacent segment disease after posterior lumbar fusion: a case-controlled study. J Orthop Surg Res. (2019) 14(1):216. 10.1186/s13018-019-1276-9 31311556 PMC6636144

[13] Miao D Fan M Zhang W Ma X Wang H Gao X The risk factors for low back pain following oblique lateral interbody fusion: focus on sarcopenia. J Orthop Surg Res. (2025) 20(1):171. 10.1186/s13018-025-05584-8 39962600 PMC11834252

[14] Oh KW Lee JH Lee JH Lee DY Shim HJ. The correlation between cage subsidence, bone mineral density, and clinical results in posterior lumbar interbody fusion. Clin Spine Surg. (2017) 30(6):E683–9. 10.1097/BSD.0000000000000315 28632554

[15] Wang YP An JL Sun YP Ding WY Shen Y Zhang W. Comparison of outcomes between minimally invasive transforaminal lumbar interbody fusion and traditional posterior lumbar intervertebral fusion in obese patients with lumbar disk prolapse. Ther Clin Risk Manag. (2017) 13:87–94. 10.2147/TCRM.S117063 28176906 PMC5261601

[16] He W He D Sun Y Xing Y Wen J Wang W Standalone oblique lateral interbody fusion vs. combined with percutaneous pedicle screw in spondylolisthesis. BMC Musculoskelet Disord. (2020) 21(1):184. 10.1186/s12891-020-03192-7 32293389 PMC7092594

[17] Cruz-Jentoft AJ Bahat G Bauer J Boirie Y Bruyère O Cederholm T Sarcopenia: revised European consensus on definition and diagnosis. Age Ageing. (2019) 48(1):16–31. Erratum in: Age Ageing. 2019 48(4):601. doi: 10.1093/ageing/afz046. 10.1093/ageing/afy169 30312372 PMC6322506

[18] Li H Li J Ma Y Li F Xu Z Chen Q. The effect of sarcopenia in the clinical outcomes following stand-alone lateral lumbar interbody fusion. J Back Musculoskelet Rehabil. (2021) 34(3):469–76. 10.3233/BMR-200138 33492276

[19] Park KH Chung HW Lee HD Jeon CH Koh JH Chung NS. Cage obliquity and radiological outcomes in oblique lateral interbody fusion. Spine (Phila Pa 1976). (2023) 48(22):1611–6. 10.1097/BRS.0000000000004507 36255377

[20] Pahor M Manini T Cesari M. Sarcopenia: clinical evaluation, biological markers and other evaluation tools. J Nutr Health Aging. (2009) 13(8):724–8. 10.1007/s12603-009-0204-9 19657557 PMC4312657

[21] Yao L Petrosyan A Fuangfa P Lenchik L Boutin RD. Diagnosing sarcopenia at the point of imaging care: analysis of clinical, functional, and opportunistic CT metrics. Skeletal Radiol. (2021) 50(3):543–50. 10.1007/s00256-020-03576-9 32892227

[22] Kotheeranurak V Jitpakdee K Lin GX Mahatthanatrakul A Singhatanadgige W Limthongkul W Subsidence of interbody cage following oblique lateral interbody fusion: an analysis and potential risk factors. Global Spine J. (2023) 13(7):1981–91. 10.1177/21925682211067210 34920690 PMC10556923

[23] Abbushi A Cabraja M Thomale UW Woiciechowsky C Kroppenstedt SN. The influence of cage positioning and cage type on cage migration and fusion rates in patients with monosegmental posterior lumbar interbody fusion and posterior fixation. Eur Spine J. (2009) 18(11):1621–8. 10.1007/s00586-009-1036-3 19475436 PMC2899391

[24] Chang S Xiang HF Wei JH Liu Y. Analysis of factors impacting inter-body fusion cage subsidence following an oblique lateral interbody fusion (OLIF) stand-alone procedure. J Back Musculoskelet Rehabil. (2025) 38(2):383–93. 10.1177/10538127241301673 39973245

[25] Kim MC Chung HT Cho JL Kim DJ Chung NS. Subsidence of polyetheretherketone cage after minimally invasive transforaminal lumbar interbody fusion. J Spinal Disord Tech. (2013) 26(2):87–92. 10.1097/BSD.0b013e318237b9b1 23529151

[26] Hu Z He D Gao J Zeng Z Jiang C Ni W The influence of endplate morphology on cage subsidence in patients with stand-alone oblique lateral lumbar interbody fusion (OLIF). Global Spine J. (2023) 13(1):97–103. 10.1177/2192568221992098 33685261 PMC9837506

[27] Hou Y Luo Z. A study on the structural properties of the lumbar endplate: histological structure, the effect of bone density, and spinal level. Spine (Phila Pa 1976). (2009) 34(12):E427–33. 10.1097/BRS.0b013e3181a2ea0a 19454994

[28] Matsuo S Kawakami M Minetama M Nakagawa M Teraguchi M Kagotani R Clinical features of sarcopenia in patients with lumbar spinal stenosis. Spine (Phila Pa 1976). (2020) 45(17):E1105–10. 10.1097/BRS.0000000000003498 32205696

[29] Sun K Zhu H Huang B Li J Liu G Jiao G MRI-based central sarcopenia negatively impacts the therapeutic effectiveness of single-segment lumbar fusion surgery in the elderly. Sci Rep. (2024) 14(1):5043. 10.1038/s41598-024-55390-1 38424180 PMC10904385

[30] Singhatanadgige W Sukthuayat A Tanaviriyachai T Kongtharvonskul J Tanasansomboon T Kerr SJ Risk factors for polyetheretherketone cage subsidence following minimally invasive transforaminal lumbar interbody fusion. Acta Neurochir (Wien). (2021) 163(9):2557–65. 10.1007/s00701-021-04923-y 34297205

[31] Kalinkovich A Becker M Livshits G. New horizons in the treatment of age-associated obesity, sarcopenia and osteoporosis. Drugs Aging. (2022) 39(9):673–83. 10.1007/s40266-022-00960-z 35781216

[32] Hou Y Yuan W. Influences of disc degeneration and bone mineral density on the structural properties of lumbar end plates. Spine J. (2012) 12(3):249–56. 10.1016/j.spinee.2012.01.021 22366078

[33] Palepu V Helgeson MD Molyneaux-Francis M Nagaraja S. The effects of bone microstructure on subsidence risk for ALIF, LLIF, PLIF, and TLIF spine cages. J Biomech Eng. (2019) 141(3):031002. 10.1115/1.4042181 30516247

[34] Jones C Okano I Salzmann SN Reisener MJ Chiapparelli E Shue J Endplate volumetric bone mineral density is a predictor for cage subsidence following lateral lumbar interbody fusion: a risk factor analysis. Spine J. (2021) 21(10):1729–37. 10.1016/j.spinee.2021.02.021 33716124

[35] Girardi FP Parvataneni HK Sandhu HS Cammisa FP Jr Grewal H Schneider R Correlation between vertebral body rotation and two-dimensional vertebral bone density measurement. Osteoporos Int. (2001) 12(9):738–40. 10.1007/s001980170049 11605739

[36] Muraki S Yamamoto S Ishibashi H Horiuchi T Hosoi T Orimo H Impact of degenerative spinal diseases on bone mineral density of the lumbar spine in elderly women. Osteoporos Int. (2004) 15(9):724–8. 10.1007/s00198-004-1600-y 14997287

[37] Zou D Sun Z Zhou S Zhong W Li W. Hounsfield units value is a better predictor of pedicle screw loosening than the T-score of DXA in patients with lumbar degenerative diseases. Eur Spine J. (2020) 29(5):1105–11. 10.1007/s00586-020-06386-8 32211997

[38] Shen S You X Ren Y Ye S. Risk factors of cage subsidence following oblique lumbar interbody fusion: a meta-analysis and systematic review. World Neurosurg. (2024) 183:180–6. 10.1016/j.wneu.2023.12.110 38145652

